# Risk Communication and Community Engagement (RCCE) implementations to control cholera outbreak in Oromia region, Ethiopia

**DOI:** 10.1186/s41182-024-00679-0

**Published:** 2025-01-13

**Authors:** Dabesa Gobena, Esayas Kebede Gudina, Getahun Fetensa, Tizta Tilahun Degfie, Tessema Debela, Afework Tamiru, Zenebu Begna Bayissa, Dereje Diriba, Tarekegn Sarbessa, Daniel Bekele, Natinel Teferi, Achalu Layesa, Abate Zewdie, Dawit Worku Ayele, Meron Debebe Mersha, Chala Bafikadu, Senahara Korsa Wake, Lemi Abebe, Tesfaye Kebebew, Tefera Goshu, Birhanu Kenate, Yadeta Dessie, Zeleke Mekonnen

**Affiliations:** 1 Public Health Emergency Management and Health Research Directorate, Oromia Health Bureau, Addis Ababa, Ethiopia; 2 School of Medical Laboratory Sciences, Institute of Health, Jimma University, Jimma, Ethiopia; 3 Department of Internal Medicine, Jimma University Institute of Health, Jimma, Ethiopia; 4 Department of Health Behavior and Society, Faculty of Public, Jimm Medical Center, Jimma University, Jimma, Ethiopia; 5 Department of Nursing, School of Nursing and Midwifery, Institute of Health Sciences, Wollega University, Nekemte, Ethiopia; 6 Department of Reproductive Health and Population Studies, Bahir Dar University College of Medical and Health Sciences, Bahir Dar, Ethiopia; 7 Department of Public Health, College of Medicine and Health Sciences, Wollega University, Nekemte, Ethiopia; 8 Department of Public Health, College of Medicine and Health Sciences, Ambo University, Ambo, Ethiopia; 9 Department of Statistics, College of Natural and Computational Sciences, Ambo University, Ambo, Ethiopia; 10 School of Public Health, College of Medicine and Health Science, Haramaya University, Harar, Ethiopia

**Keywords:** Cholera, Community engagement, Ethiopia, Risk communication, Outbreak, Oromia

## Abstract

**Background:**

Oromia regional state experiencing cholera outbreaks in a protracted pattern despite various interventions at local and regional levels. This study aimed to examine the implementation of Risk Communication and Community Engagement (RCCE) activities for cholera outbreak control in the region.

**Methods:**

We conducted a quantitative and qualitative mixed-method study. The study included 422 respondents for quantitative, 22 key informant interviews (KII), and 4 Focus Group Discussions (FGDs) for the qualitative methods. Risk Communication and Community Engagement (RCCE) activities were assessed using standard questionnaires adapted from national cholera guideline later categorized as poor, satisfactory and good. The findings have also been derived qualitatively from three distinct themes or pillars, specifically (coordination and logistics, RCCE, and the Oral Cholera Vaccine). The quantitative data were analyzed using Stata, version 14.0, and ATLAS.ti9 software was used for qualitative data analysis. An ordinal logistic regression model was applied to identify factors associated with the RCCE status, and a thematic content analysis was performed for the qualitative study. Odds Ratios with 95% confidence intervals (CI) were used to present the findings from the quantitative analysis.

**Results:**

Only 53% (223) of participants had received health information on cholera of whom 22.8% (96) had material for Social Behaviour Change (SBC) in the local language (Afan Oromo). The overall RCCE implementation status was rated as poor by 73% of the respondents, satisfactory by 23%, and only 4% rated it as good. Level of education and occupation of the house are among the factors affecting the implementation of RCCE. The qualitative findings revealed a lack of regular community dialogues, and community engagements were notably minimal during the early phase of the outbreak. Overall, the RCCE implementation activities were characterized by inconsistency, a lack of comprehensiveness, and uniformity across all levels.

**Conclusion:**

The RCCE-related intervention activities were found to be minimal, inconsistent and less focused. The RCCE interventions and awareness creation need to begin with the small units of the community structures, including individuals and families and have to happen continuously with the community, and health workers' involvement at all level. Preliminary evaluation of Social and Behaviour Change (SBC) materials before their distribution should be made, and adopting diverse communication modalities to control the outbreak.

## Introduction

Cholera outbreaks remain to continue worldwide causing significant mortality and morbidity [1–4]. The current status of cholera outbreaks, as reported by the World Health Organization (WHO), indicates a significant resurgence of the disease globally. In 2023, over 667,000 cases and 4,000 deaths were recorded, marking an increase from the previous year [5]. The outbreaks are concentrated in at least 30 countries, particularly in the WHO African Region, with the Democratic Republic of Congo, Zimbabwe, and Sudan experiencing high case rates [5, 6]. The outbreaks lead to loss of life and halt socioeconomic development grossly. In 2016, 38 countries reported a total of 132 121 cases, including 2420 deaths, resulting in an overall case fatality rate (CFR) of 1.8% [2]. Cholera is a stark marker of inequality, disproportionately affecting the poorest and most vulnerable populations around the world and within each affected country [1].

The African continent experiences a disproportionately high burden of cholera cases and cholera-related deaths as they are still far behind in their WASH (Water hygiene and sanitation) status which is a favorable situation for the disease [3–5]. From 2000 to 2015, 83% of cholera deaths reported by the WHO occurred in sub-Saharan Africa [3]. Evidence indicates that these figures could be higher if social, political, and economic disincentives for reporting cholera cases are considered. It remains a persistent health problem in sub-Saharan Africa and worldwide [7]. Recurring cholera outbreaks indicate deprived water and sanitation conditions and weak health systems, contributing to the transmission and spread of the cholera infection [8]. In Ethiopia over 15.9 million people live in cholera-prone areas, with annual outbreaks reported [9]. From 2001 to 2023, there were 215,205 cholera cases and a cumulative case fatality rate (CFR) of 1.10% [10]. The 2022 outbreak recorded 841 cases with a CFR of 3.13%, while 2023 saw over 30,000 cases and a CFR of 1.4% [9].

In the Oromia region of Ethiopia, cholera outbreaks have been occurring every year but have started to affect the communities in large numbers since August 28, 2022 (Oromia cholera outbreak line list admin report). The regional government initiated an outbreak response incorporating all pillars, including the RCCE to combat the outbreak. Through this RCCE, the patient and the family are expected to be educated about personal hygiene, boiling water, improving sanitation, and avoiding undercooked seafood and raw fruits and vegetables [11]. Frequent communications are expected since outbreaks are frequently marked by uncertainty, confusion, and an increased sense of urgency [12]. Thus, this study aims to evaluate the implementation status of RCCE activities during the 2022/2023 cholera outbreak in the Oromia region of Ethiopia. The study employed concurrent qualitative and quantitative data collection of the cholera RCCE implementation activities in the middle of the cholera outbreak in the defined areas.

## Methods

### Study design and setting

A cross-sectional study with both qualitative and quantitative methods was conducted in two zones of the Oromia regional state of Ethiopia from July 23 to August 10, 2023. The study area covers the Gedeb Assasa district of West Arsi Zone, Moyale District, and Moyale Town of Borena Zone. Gedeb Assasa is located about 277 km to the southeast of Addis Ababa. According to the 2007 national census report, the district had a total population of 187,799, with 92,471 males and 94,887 females. Most of the population of the woreda, 167,152 (89%), were rural residents [10]. The district has no general hospitals, 08 health centers, and 25 health posts that provide health services to the community.

Moyale district is one of the woredas of the Borena Zone bordering Kenya, with administrative structures based in Moyale town. Moyale town is the district with the leading market center situated about 771 km south of Addis Ababa, connecting Ethiopia with Kenya with the main road crossing the country. According to the 2007 national census, the district had a population of 31,158; of them, 16,127 were males, and 15,031 were females; all of them were rural residents, while another 28,056 inhibits were classified as urban, with a special enumeration (CSA, 2008). The area was recently restructured, and Moyale town was newly established as an independent administrative structure separate from the district. The district and town have 23 and 13 health facilities, respectively, providing health care services. This study used a mixed approach, combining qualitative and quantitative techniques in the form of triangulation, to back the qualitative aspects with numerical information.

### Source and study population

All residents of the selected kebeles and stakeholders engaged in the cholera outbreak response were considered as the source populations. The study population consisted of selected household members, health facilities across various tiers, and other supportive government office at different levels that are partaking in the cholera epidemic response. The inclusion criteria for the study were 18 years and above, and a permanent resident of the selected kebeles.

### Sample size determination

A single population proportion approach was used to determine the sample size for the household survey, considering a 50% prevalence of RCCE implementation, a 95% confidence interval, and a 5% marginal error. Accordingly, the total calculated sample size was 422, accounting for 10% of the non-response rate.

### Sampling procedure and sampling techniques

Among selected districts, there was a random selection of kebeles by considering 30% of kebeles of each district, and a proportional allocation of the final sample size was made for each kebele. The final households were selected through systematic sampling.

**Fig. 1:**
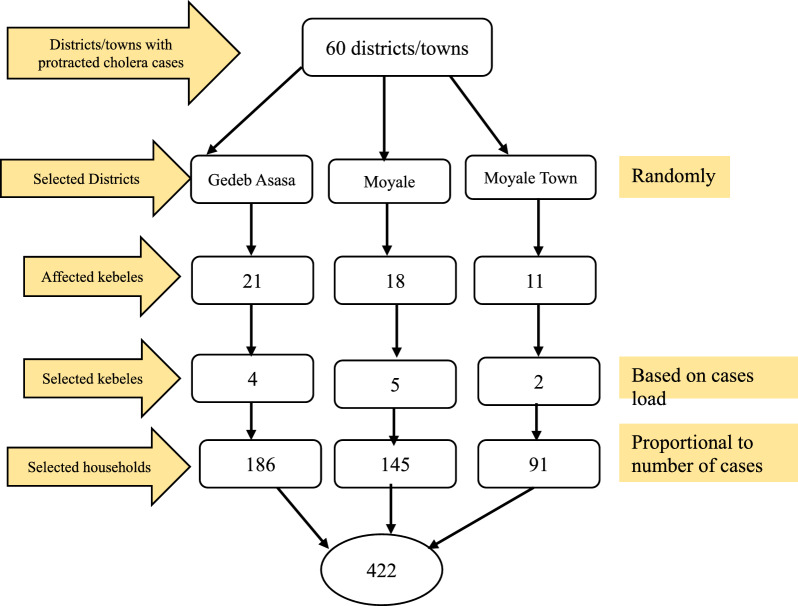
Schematic presentation of sampling procedure and techniques

Three districts were randomly selected using a lottery method from a list of sixty districts experiencing a prolonged cholera outbreak. Subsequently, within each district, 30% of kebeles were identified based on the prevalence of the cases. Finally, households were selected in proportion to the number of cases documented in the chosen kebeles through systematic sampling (Fig. 1). Participants for the qualitative methods were purposely selected and interviewed.

### Data collection tools and procedure

This study used both primary and secondary data sources. The secondary information was mainly obtained from written records and archived materials. The primary data were collected by employing multi-level data collection techniques. The combination of methods is believed necessary to enrich the research by diversifying the sources of information; to achieve the aim of triangulation, where findings of one method will be checked/supported against those of others; to benefit from the strengths of each technique and to compensate for the weaknesses; and to establish confidence and obtain trustworthiness of the research findings [11, 12]. The primary data were collected by employing multi-level data collection techniques. Primary data collection tools for this study were the household survey, Key informant interview (KII), Focused Group Discussion (FGD), and Observation.

### Household survey

A household survey was used to capture quantitative data on respondents' demographics and clinical characteristics. A standard survey questionnaire was employed to collect the information from 422 selected households using electronic tablets. Eight experienced data collectors collected the data. We trained them on the objectives of the study, the data collection tools, and the data collection software. The survey tool was translated from English into widely spoken local languages (Afaan Oromo) for the data collection.

Trained supervisors checked the work of data collectors daily using the online CSentry CSPro Data Entry 7.2.1 application software for completeness and data quality. The data were sent to the central server, and the data's completeness was checked immediately. The field supervisor was responsible for ensuring that data collectors followed the protocol to correct any errors in completing the questionnaires early. In case of data that need verification, for example, vaccination status, the interviewer observed an immunization card or visited the nearest health facility to review records of that particular individual. Further, the questionnaires were pre-tested in Sheshemene and Yabello towns on 5% of the sample size for appropriateness and to validate the data collection tools.

### Key informant interview

Key informant interviews (KII) were done with informants chosen from the health systems and officials at various levels involved in the outbreak response. An in-depth interview was conducted between 24 and 122 min with 18 key informants drawn from the two study zones. These informants included representatives of zonal, district, and kebele administrations; zonal and district PHEMs and heads of health facilities; zonal and district water and energy bureaus; and health extension workers from both sites. The interview guide adopted from WHO was used, but questions were only sometimes ordered and worded the same way, allowing for different follow-up and probing questions. Selected epidemiologists and researchers from Jimma University, Ambo University and Mada Walabu University conducted both the interview and FGD.

### Focus group discussion (FGD)

The FGD was used to have group consensus and opinions on health system preparedness, implementing risk communication, and community engagement to address the cholera outbreak. It was also used to understand people's experiences with risk communication and their participation in controlling the epidemic. Four focus group discussions, each consisting of between 8 and 13 participants, were held in Moyale and Gedeb Assasa towns. While the two FGDs were held with groups of community representatives (one in each town), the remaining two were conducted with staff working in cholera treatment centers (one in each town). Accordingly, a total of 39 informants took part in the FGD. The same guiding questions guided the FGD as the key informant interviews. The discussion was audio recorded and held in the local language (Afan Oromo). The FGDs generally last between 67 and 139 min.

### Observation

Observations of the intervention activities and the surrounding area were made during the data collection. This made it possible to understand discrepancies between the information gathered and the actual phenomena on the ground. It helped to gather practical phenomena to supplement the data gathered through surveys, interviews, and focus group discussions. In this regard, an attempt was made to observe waste management and disposal practices, cholera treatment centers (CTC), water points, and banners and billboards intended to indicate the risk of the outbreak. Some were photographed to provide further illustrations and explanations of the context.

### Data analysis

The quantitative data stored on the central server were exported to Microsoft Excel and then to the statistical software Stata version 14.0, which was used for data clearing and analysis. The result was analyzed by each pillar (surveillance, case management, water sanitation, hygiene, and social mobilization), and a composite variable was developed from each pillar to assess the cholera response implementation status at the household level. However, for this manuscript, the composite variable developed from social mobilization was used to assess the overall implementation status of RCCE at the household level. The cholera response implementation status order was rated as ‘Good' if the respondent rate for each composite variable by pillar was greater than 75%, 'Satisfactory' if 50–75%, and 'Poor' if they scored less than 50%. Finally, the overall response intervention status was assessed by summing the composite variables created by the pillars. The result was summarized with absolute and relative (percentage) frequencies. Factors associated with the RCCE implementation were examined using ordinal logistic regression. The odds ratio from the ordinal logistics regression model with point and interval (95% confidence intervals, CI) estimates were used to test the association between the response implementation and exposures. A p-value less than 0.05 was considered statistically significant.

For the qualitative study, the audio-recorded data were transcribed to the English version, coded, and categorized to generate three distinct themes or pillars, specifically (coordination and logistics, RCCE, and the Oral Cholera Vaccine) using Atlas ti9. Then, the qualitative findings were triangulated with the statistically analyzed results.

### Conceptual and operational definitions

#### Implementation status

Well, implemented/good: If the cumulative sum of variables under each pillar will score greater or equal to 75% or 4 and 5 out of five responses.

Satisfactory: If the cumulative sum of variables under each pillar will score 50–75%.

Poor: If the cumulative sum of variables under each pillar will score less than 50%.

Preparedness: The process of ensuring readiness for a cholera outbreak in advance to make the response more effective.

Case management: (case management quality, re-hydration therapy according to protocol, preventive measures at home and CTC (isolation, hygiene), trained workforce, the emergency stock of supplies, infection prevention and control measures observed).

## Results

### Findings from the quantitative survey

#### Brief demographic characteristics

This study included 422 households. Three hundred fourteen (74.4%) respondents were wives, whereas 80 (19.0%) and 28 (6.6%) were husbands and other family members of the household, respectively. Respondents' mean age was 36.7 years (Table 1).

**Table 1:** Socio-demographic characteristics of respondents, cholera outbreak household survey, Oromia region, Ethiopia, 2023

Variable	Category	Count (*n* = 422)	Percent
Respondent	Wife	314	74.4
	Husband	80	19.0
	Other family members	28	6.6
Residence	Rural	293	69.4
	Urban	129	30.6
Marital status	Single	23	5.5
	Married	354	83.9
	Widowed	33	7.8
	Divorced	12	2.8
Father	No formal education	166	39.3
	Can read and write	28	6.6
	Primary (grade1-8)	104	24.6
	Secondary (grade 9–12)	53	12.6
	Diploma and vocational	15	3.6
	Degree and above	17	4.0
	N/A(Died)	39	9.2
Mother's education status	No formal education	251	59.5
	Can read and write	23	5.5
	Primary (grade1-8)	87	20.6
	Secondary (grade 9–12)	31	7.3
	Diploma and vocational	7	1.7
	Degree and above	11	2.6
	N/A (died)	12	2.8
Household head occupation status	Farmer	163	38.6
	Pastoralist	25	5.9
	Housewife	4	0.9
	Daily laborer	90	21.3
	Government employee	60	14.2
	NGO employee	3	0.7
	Merchant	56	13.3
	Other (specify)	21	5.0

#### Overall implementation of RCCE

To assess the overall RCCE implementation at the home level, the accessibility, clarity, and content of health information received as part of the outbreak response were evaluated. As a result, 53% (223) of participants had received health information on cholera. Of the participants, 22.8% (96) had received material for Social Behaviour Change (SBC) in Afan Oromo. While only 25% (106) received instructions on preparing ORS at home, only 21% of all study participants correctly used chlorinated water for drinking water (Table 2).

**Table 2:** Implementation of RCCE activities at the household level in three active cholera outbreak districts of Oromia, Ethiopia, 2023

Variable	Category	Frequency (%)
Health information/education	Yes	223 (52.8)
Handwashing habit	Yes	190 (45.0)
proper use of chlorinated water	Yes	88 (20.9)
Experience in making an ORS or homemade solution	Yes	106 (25.1)
Prompt recognition of symptoms and referral	Yes	102 (24.2)
Had experience with safer excreta disposal	Yes	107 (25.4)
Practiced hygienic food preparation and storage	Yes	134 (31.8)
Don’t eat raw food/undercooked food	Yes	27 (6.4)
Sources of information about cholera (*n* = 223)	HEW and HW	208 (93.3)
	Peers, religious leader	10 (4.5)
	Media	5 (2.2)
Availability of materials for SBC in Afan Oromo	Yes	96 (22.8)
The message provided is straightforward	Yes	180 (42.7)
Attended any in-home health education sessions	Yes	65 (15.4)
Attended hygiene promotion campaign	Yes	14 (3.3)
Take part in cholera discussions in the community	Yes	78 (18.5)
Households who are aware of the ORP/ORS corner exits	Yes	92 (21.8)
Attended demonstration/sensitization how to use ORS	Yes	89 (21.1)
HEW in the last 6 months visit	Yes	244 (57.8)
HEW visit	> Month	74 (30.3)
	< Month	170 (69.7)

#### Knowledge of cholera

Even though only 2.3% of the respondents to the survey claimed to have cholera, 99% of respondents (420) had heard of the illness. Ninety-three percent (394) of individuals claimed they were aware of the means of cholera transmission. With regard to identifying signs and symptoms of cholera, respondents predominantly cited watery diarrhea 98.3% (398), vomiting 95.30% (386), and generalized body weakness 6.4% (26) while 5.7% (23), and 4.2% (17) still claimed incorrect symptoms, fever, and headache, respectively.

In line with the quantitative finding, field researchers also observed that many local informants at both sites were aware of the disease. They explicitly stated that it dehydrates the body and kills in a short period. This increased awareness was achieved because, first, in the case of Moyale, the disease had been recurrently experienced. Thus, residents became more familiar with the disease's causes, symptoms, and consequences. Second, even though cholera had not previously been experienced in Gedebe Hassasa, many people have come to learn about the disease due to the public awareness created in the area.

#### Community action to suspected cholera cases at the household level

Among study participants, 25.4% (107) said they administer ORS at home for cholera cases. Furthermore, 30.8%, 5.7%, and 69.5% of poll respondents stated they would treat suspected cholera with a mixture of sugar and salt, isolate the patient at home, and contact the nearest medical center, respectively. In comparison, 4.5% (19) said they would pray, and all participants apply holy water. Most respondents, 69.9% (295), responded that most of the suspected cholera cases in the area were taken to a public health facility (Table 3).

**Table 3:** Community-level action taken for suspected cholera cases in selected districts of Oromia region, Ethiopia, 2023

Variable	Category	Frequency (%)
Actions taken at home for suspected cholera		
Administer ORS at home	No	315 (74.6)
	Yes	107 (25.4)
Treat by mixing sugar and salt	No	292 (69.2)
	Yes	130 (30.8)
Keep the person alone	No	398 (94.3)
	Yes	24 (5.7)
Pray	No	403 (95.5)
	Yes	19 (4.5)
Inform the health facility	No	127 (30.1)
	Yes	295 (69.9)
Actions taken outdoors for suspected cholera		
Go to a government health facility	No	12 (2.8)
	Yes	410 (97.2)
Go to a private health facility	No	410 (97.2)
	Yes	12 (2.8)
Other	No	417 (98.8)
	Yes	5 (1.2)

#### Factors associated with cholera outbreak RCCE at the household level

Ordinal logistic regression was adopted to identify factors associated with RCCE implementation. Factors such as the respondent's age, family size, household head's occupation, father's educational level, mother's educational level, other family members' educational levels, and residential location were considered in this study. The findings showed that factors like the education of fathers, household head occupation, and age of respondents significantly affected the RCCE implementation status of respondents. A positive coefficient of these factors indicates that a respondent with a higher score on the independent variable is more likely to be observed in a higher category of the dependent variable. At the same time, a negative coefficient indicates the chances that a respondent with a higher score on the independent variable will be observed in a lower category of the dependent variable. Consequently, the odds of the implementation of RCCE were 2.37 times higher in households characterized by a low level of education in comparison to those with secondary education or higher. In a similar vein, the odds of RCCE implementation were 0.21 times greater among households whose father’s occupation was pastoralist as opposed to those with members in formal employment (Table 4).

**Table 4:** Factors associated with the implementation of RCCE at the household level

Parameter	Category	AOR	*P*-value	95% CI (AOR)	
				Lower	Upper
Respondent	Wife	Reference			
	Husband	0.67	0.24	0.34	1.31
	Other	0.17	0.11	0.02	1.46
Education of father	No formal education	1.26	0.57	0.56	2.86
	Can read, write and primary	2.37	0.04	1.03	5.43
	Secondary and above	Reference			
Education of mother	No formal education	0.55	0.19	0.22	1.35
	Can read, write and primary	0.78	0.59	0.31	1.94
	Secondary and above	Reference			
Education of other	No formal education	0.99	0.96	0.58	1.69
	Can read, write and primary	1.14	0.75	0.52	2.47
HH head	Secondary and above	Reference			
	Wife				
	Husband	1.11	0.78	0.52	2.39
HH head occupation	Government employee	Reference			
	Merchant	0.64	0.30	0.28	1.48
	daily laborer	0.69	0.35	0.31	1.51
	Farmer, pastoralist and housewife	0.21	< 0.0001	0.10	0.46
	Other	0.24	0.03	0.07	0.86
Age	18–24	Reference			
	25–34	1.98	0.11	0.86	4.59
	35–44	2.07	0.13	0.80	5.31
	> 45	2.59	0.05	1.02	6.58
		Reference			

### Findings for the qualitative study

#### Risk behavior facilitating the cholera transmission

The risk behaviors facilitating the transmission of the outbreak in both sites included open defecations, the consumption of poor-sanitized water and foods (on the street or from restaurants), and the problem of waste disposal. As detailed below, in Moyale, the problem of toilets and open defecation, mainly using plastic bags and plastic bottles, was common. In addition, children have collected plastic bottles that have already been thrown away with urine to use for drinking water. They split the urine from the plastic bottles, refilled them with water from 'ela' (water point), sold it to restaurants, and then sold it to food consumers. Often, restaurant customers have yet to learn about its quality but prefer this water to bottled water due to its cheap price. This can be elaborated by quotes from qualitative participants as follows:

Though not explicitly stated by residents, open defecation was also practiced in Gedeb Hassasa, promoting transmissions. As the data from Hassasa Health Centre indicated, the health center served seven catchment kebeles; four were declared open defecation-free, while the remaining three were not free from open defecation.

On both sites, some different fruits and vegetables have been sold on the streets and are accessible to many people. Foods such as boiled potatoes, pie (locally called 'ambasha'), and fried homemade biscuits have been on the roadside and consumed by many individuals. These foods are easily contaminated and being served and eaten raw and/or with poor sanitation due to a water shortage in Moyale, as confirmed in Gedeb Hassasa.

In this regard, an elder FGD participant stated that "*we should speak the truth. I want to speak the truth. People are facing a big problem with inflation. This time, the poor cannot buy a single biscuit, which costs 15 birr. A poor man may buy after going 2 days without eating…*”.

#### Risk communication and preparedness

During data collection, the research team verified that there was one risk communication signboard in Moyale town but none in Hassasa town. In this regard, the best practice was that the health center in Moyale made a post-awareness campaign survey about the understandability of risk communication. Still, a mechanism has yet to be set up for such feedback in Gedeb Hassasa.

Risk communication activities were conducted, mainly through the campaign. According to the local informants, risk communication and awareness about the epidemic were mainly communicated orally using sound amplifiers in marketplaces during market days and public gatherings, such as religious places. The informants indicated this public communication was in three different languages: Afan Oromo, Amharic, Afan Somali in Moyale, and Afan Oromo in West Arsi. However, the survey result revealed that only 96 (22.7%) household respondents reported that Social Behaviour Change (SBC) materials were available in Afan Oromo, and most respondents, 57.4% (242), stated that the message needed to be more complex to understand. This has created difficulty in understanding and a need for more attention to the message. This challenge was further exacerbated by the limited attempts to employ alternate communication modalities. In this regard, using flyers, brochures, and signboards was limited or unavailable at both sites. An elder participating in FGD, for instance, explained the lack of visual communication but its value in influencing behavior, saying,

"There is an announcement using an ambulance, but posters are not yet implemented. There should be a pictorial representation of what a person infected with cholera looks like. It will be quite helpful. The last time I saw the Red Cross showing how the disease was manifesting, We were all worried after seeing the pictures. Such things are too important to awaken the community. This should be done soon. Though many things have been done, I have not seen such visual communication in this town" (FGD with community representative Gedeb Hassasa, 1 August 2023).

During the initial stage, the message needed to be understood and accepted by the community at the study sites. In this regard, the post-campaign survey assessment in Moyale indicated that more than 60% did not understand and were negligent of the risk communication messages. This negligence was partly related to the communication barrier and the transitory nature of many of the residents in the area. Many were temporary dwellers; they moved in and out for jobs between the bordering areas. Some of these groups, such as daily laborers, come from different language origins and have often faced language barriers and difficulty understanding communication in Moyale. Further, many permanent residents did not pay attention and were reluctant to accept the message during the initial period due to preexisting diarrhea disease. Just before the onset of the cholera epidemic, there were prevalent diarrhea diseases in Moyale, and the community considered the message irrelevant and as if it were being repeated to inform them about the already existing disease.

In Gedeb Hassasa, the health extension workers (HEWs) were deployed to communicate risks and provide awareness. However, the community was reluctant to listen to health extension workers' advice and preferred other professionals or officials. It was mainly because they (the local community) felt that the HEWs were not competent enough and had better knowledge about the outbreak than the local people.

Furthermore, the failure to accept the message at study sites was also related to the impracticality of the message, where adequate and safe water was unavailable. Still, the message urged them to use safe water. During the fieldwork, the creation of risk communication and awareness, mainly advice, was considered impracticable given the poor infrastructure and facilities such as water, waste management systems, and sanitation. Many local informants mocked the officials, as they were not concerned with saving the community but just spending the budget. Thus, the creation of risky communication and awareness was perceived by some community members as a waste of money.

#### Community engagement and participation

There had been attempts to involve the community in risk communication and support the effort to contain the outbreak. However, despite the possibility of coordinating and engaging some community members, community engagement could have been improved during the initial period at the study sites. Furthermore, it was noted that there was no equal participation among different segments of the community in both areas.

In Gedeb Hassasa, elders and religious leaders engaged in communication and awareness creation. At the same time, some economic elites of the community also participated and supported the awareness campaign financially and materially. In this regard, with the participation of NGOs and the local economic elites, the west Arsi zone managed to mobilize birr 17 million to respond to the outbreak. The involvement and participation of religious figures in risk communication could also be the best practice in the zone. The religious leaders provide risk communication and awareness for their churches and mosque attendees, mainly at the end of their regular religious service.

Further, instrumentalizing 1–10 development networks was paramount to mobilizing and engaging the local community members towards awareness creation as well as overall activities to respond to prevent and control the outbreak in the area. In Moyale, the WaSH club was established, and the mini-media was used to communicate the risk to students. The school's WaSH club had about 10 student members who actively communicated messages to the school community through dramas and poems and to parents on their way home. Furthermore, voluntary students who were members of the Red Cross were used to educate about water treatment chemicals (such as Aqua Tap) through practical demonstrations on how to use them. This was also mentioned as a best practice to inform and reach the parents.

## Discussion

The overall implementation status of RCCE for this study was evaluated, as poor for 73% satisfactory for 23% and 4% as good. This contradicts the recommendation made by Holmgren J et,al which states that, risk communication and community engagement are crucial during an outbreak to prevent and control it [1]. Risk Communication and Community Engagement can be achieved through the involvement of government structures from district, zone/regional, and federal levels in availing adequate and timely support with technical expertise, supplies, resources, situation analysis, decision-making, communications and reporting [13].

The study results show minimal engagement activity, as only 53% (223) of participants had received health information on cholera. This differs from the study conducted in Addis Ababa, which revealed that exposure to cholera-related messages and outbreak information was 71.8% and 52.7%, respectively. This may be due to the study setting differences as the current study was conducted among the rural community [2] and there were delays in sharing case information that impeded this approach's rapid implementation. At the same time, evaluation of the effectiveness of interventions varied [14]. This is because the cholera outbreaks in the African Region, including in Ethiopia, occur in the context of natural disasters. Poor sanitation and unreliable water supplies with increased cross-border movements also drive the outbreak across the region [15]. It is essential to consider a diversified strategy to control cholera and lower its mortality rates [16]. People actively participate in controlling the cholera outbreak by promoting safe, healthier practices, facilitating community action, and helping to reduce fear, stigma, and misinformation [17]. Ensure appropriate RCCE planning, resourcing, coordination, management, and listening structures are established at national and local levels to ensure affected and at-risk communities are engaged, informed, and included in planning and implementing all relevant outbreak readiness and response components. Create an enabling environment and disseminate Risk Communication and Community Engagement messaging in a timely and appropriate manner through trusted channels to encourage the uptake of preventative, protective, and care-seeking behaviors. Responding to rumors and misinformation through proper communication channels accessible and trusted by at-risk communities is crucial during any outbreak [18].

The results demonstrated that variables such as the educational attainment of fathers, the occupational status of household heads, and the age of respondents had a significant association with the implementation status of RCCE among the respondents. In this regard, fathers possessing literacy skills and having completed elementary education exhibited a higher likelihood of engaging in the implementation of RCCE compared to those who had attained secondary education or higher. Similarly, it was observed that farmers were more actively involved in the implementation of RCCE than their counterparts employed in governmental positions. This phenomenon can be elucidated by the targeted nature of RCCE interventions at the community level, focusing on the most vulnerable and affected populations, as well as the observed lack of consistency in RCCE activities across various levels. This particular finding stands in contrast to a study conducted in Addis Ababa and Bangladesh [3].

The result reveals that the current study area's overall implementation status of the cholera outbreak response is inadequate. This indicates that coordination among actors and integration between sectors is not accessible during outbreak control [14]. However, there was considerable community engagement using different methods, including languages and SBC. Engaging in systematic Focus Group Discussions (FGDs) serves to evaluate the efficacy of diverse communication channels, methodologies, and linguistic frameworks employed to connect with distinct demographic groups, thereby ensuring that the conveyed information is not only received, but also comprehended, trusted, and deemed valuable [15].

The implementation status of cholera control and containment has been determined, among other things, by effective social mobilization and risk communication. In this regard, various interventions have been carried out in the research area to ensure the implementation of RCCE. These endeavors had both significant strengths and shortcomings. The substantial part of the intervention includes: initially, an endeavor was undertaken to render the risk communication message comprehensible and to engage community members in activities aimed at fostering awareness. Therefore, some of the intervention's strengths were mobilizing, engaging, and working with community notables such as local elders, religious leaders, and economic elites, as well as local institutions such as 1–10 development networks [19–24]. There were also attempts to get feedback about the understandability of the risk communication message, although this was restricted in Moyale. The post-campaign survey in Moyale is one such feedback-gathering tool that could be mentioned as the intervention's strength.

On the other hand, some of the shortcomings of the intervention include the following: risk communication relies significantly on oral communication in public places, but other modes of behavioral change communication, such as visual and pictorial, are less widely used. The material for changing social behaviors was also not fully developed in the local language, nor was the message straightforward. This creates a difficult understanding and a lack of interest in highlighting the risk communication messages [25–27]. Furthermore, there was no well-established mechanism to receive feedback on risk communication, though an attempt was made in Moyale. This made it difficult to promptly take corrective action in case of any challenges related to understanding the behavior-changing messages. The main limitation of this paper is the insufficient coverage of the extensive geographic region impacted by the prolonged nature of the cholera epidemic, attributable to the seasonal timing of data collection during the summer, as well as limitations related to operational budgetary considerations, logistical challenges, and human resource availability.

### Strength and limitation of the study

The study considered mixed method which was used to capture both contextual and quantitative events used to control cholera outbreak. The study also used to collect data by using variety of data collection methods, which can overcome limitation of one method of data collection and different data source which includes both primary and secondary data source was employed from three Woredas located within different administrative zones. However, the study was not free of limitation as the study was limited to insufficient coverage of the extensive geographic region impacted by the prolonged nature of the cholera epidemic, attributable to the seasonal timing of data collection during the summer, as well as limitations related to operational budgetary considerations, logistical challenges, and human resource availability.

## Conclusions and recommendations

The finding elucidated that there existed initiatives and endeavors within the study domain aimed at fostering community involvement and participation. The Social Behaviour Change materials were available, but it was difficult for many locals to access them in their native language and understand the message.

Further, no system was in place to entertain community feedback on risk communication. Likewise, the risk communication, community involvement and participation in the earlier stage were generally limited.

Locals and national stakeholders working on this response are recommended to address the problem by creating vibrant and informative resources such as e-posters and eye-catching visual documentaries with short and easy-to-understand awareness messages in their respective languages. Preparing and disseminating inclusive messages to address special populations is also essential. The involvement and active participation of community representatives such as elders, religious leaders, and notables in risk communication and activities of response should be further encouraged to enhance wide acceptance and promote behavior change in the community.

## Data Availability

No datasets were generated or analyzed during the current study.
